# Sensory Profiles in School-Aged Children with Autism Spectrum Disorder: A Descriptive Study Using the Sensory Processing Measure-2 (SPM-2)

**DOI:** 10.3390/jcm11061668

**Published:** 2022-03-17

**Authors:** Antonio Narzisi, Maddalena Fabbri-Destro, Giulia Crifaci, Stefano Scatigna, Federica Maugeri, Stefano Berloffa, Pamela Fantozzi, Adriana Prato, Rosy Muccio, Elena Valente, Valentina Viglione, Edoardo Pecchini, Susanna Pelagatti, Renata Rizzo, Annarita Milone, Rita Barone, Gabriele Masi

**Affiliations:** 1 Department of Child Psychiatry and Psychopharmacology, IRCCS Stella Maris Foundation, 56128 Pisa, Italy; stefano.scatigna@studenti.unipi.it (S.S.); stefano.berloffa@fsm.unipi.it (S.B.); pamela.fantozzi@fsm.unipi.it (P.F.); rosymuccio@yahoo.com (R.M.); elena.valente@fsm.unipi.it (E.V.); valentina.viglione@fsm.unipi.it (V.V.); edoardo.pecchini@gmail.com (E.P.); annarita.milone@fsm.unipi.it (A.M.); gabriele.masi@fsm.unipi.it (G.M.); 2 Institute of Neuroscience, National Research Council (CNR), 43125 Parma, Italy; maddalena.fabbridestro@in.cnr.it (M.F.-D.); crifaci.giulia@in.cnr.it (G.C.); 3 Child Neurology and Psychiatry Section, Department of Clinical and Experimental Medicine, University of Catania, 95124 Catania, Italy; fmaugeri@unict.it (F.M.); aprato@unict.it (A.P.); rrizzo@unict.it (R.R.); rbarone@unict.it (R.B.); 4 Department of Computer Science, University of Pisa, Largo Pontecorvo 3, 56127 Pisa, Italy; susanna@di.unipi.it; 5 Research Unit of Rare Diseases and Neurodevelopmental Disorders, Oasi Research Institute-IRCCS, 94018 Troina, Italy

**Keywords:** Autism Spectrum Disorders, sensory profile, children, Sensory Processing Measure

## Abstract

Background: Sensory reactivity is considered one of the diagnostic criteria for Autism Spectrum Disorders (ASD) and has been associated with poorer functional outcomes, behavioral difficulties, and autism severity across the lifespan. The characterization of the sensory processing in ASD has thus become crucial to identify the sensory and motor features influencing the development of personal autonomy. Objectives: The present study has two aims: (1) to compare the sensory processing between school-aged children with ASD and typically developing peers (TD); (2) to evaluate whether, within the ASD sample, the cognitive level and reported sensory symptoms explain the scores exhibited at the Sensory Processing Measure (SPM-2). Methods: The SPM-2 test was administered to the parents of 105 children with ASD and 70 TD. The ASD group was further subdivided into two groups, namely high and low functioning based on their cognitive level (High Functioning (HF), IQ > 80; Low Functioning (LF), IQ < 80). Results: ASD children exhibited higher scores throughout the SPM-2 total score and its multiple subscales. Within ASD, while HF and LF children did not differ in terms of the SPM-2 total score, a significant difference was found for the hearing, social participation, and balance and motion subscales. Conclusions: Aside from classical knowledge that the ASD population suffers from sensory processing disorders, we revealed that different sensory patterns are associated with high or low cognitive functioning. Beyond its neurobiological interest, such knowledge may be of fundamental importance for individualizing psychoeducational interventions in preschool- and school-aged children and later developmental stages.

## 1. Introduction

The presence of unusual sensory features in children with Autism Spectrum Disorders (ASD) has been consistently reported already since Kanner’s early descriptions [1], but historically they were thought to represent secondary consequences of differences in social–cognitive processing [2]. In the last two decades, several studies have indicated that atypical sensory experience occurs in as many as 90% of autistic individuals [3,4,5], with a prevalence in the typically developing population estimated between 5% and 16% [6,7]. Sensory reactivity is nowadays considered one of the diagnostic criteria for ASD and has been associated with poorer functional outcomes, behavioral difficulties, and autism severity across the lifespan. The characterization of sensory processing in children with ASD has thus become crucial to identify the sensory and motor features influencing the development of personal autonomy.

According to Miller and colleagues [8], atypical responses to sensory inputs can be classified into three major patterns, namely sensory over-responsivity (SOR), sensory under-responsivity (SUR), and sensation seeking. Concerning ASD children, clinical studies [9,10], autobiographical narratives [11,12,13], and parents’ reports [14,15] converge in indicating difficulties in regulating and organizing the type and intensity of behavioral reactivity to sensory inputs from the environment. Children with ASD often show the co-existence of more than one sensory pattern [16,17], with the highest prevalence of SUR, followed by SOR and sensation seeking [7]. These notions led in 2013 to the inclusion of both SOR and SUR among the DSM-5 diagnostic criteria for ASD (American Psychiatric Association 2013) [18], while sensation seeking was already included in DSM-IV within the rubric of unusual sensory interests [19].

The manifestations of sensory abnormalities in individuals with ASD may involve different sensory modalities. Atypicality in auditory processing constitutes the most frequently reported sensory difficulty in both hyper-sensitivity [4,20,21] and hypo-responsiveness to auditory stimuli [22,23,24,25]. Of note, the latter (e.g., decreased response to name) is described as an early index of discrimination between typically developing children and children with ASD. Considering the visual input processing, avoiding eye contact and inappropriate use of referential gaze have been widely described as early features of social deficit in ASD and associated with an internal mechanism of self-regulation that would compensate for difficulties in modulating visual inputs [14,23,26]. Finally, hyper-responsiveness to tactile inputs has also been described [9,11,27]. In some autobiographical accounts, being touched is associated with an intense sensation that can be overwhelming and disorienting [9]. Grandin [11], for example, stated that certain clothing fabrics could make her extremely anxious, distracted, and restless. ASD children with tactile hypersensitivity manifest rigid behaviors, repetitive verbalizations, and visual stereotypes [27]. Given the widespread impact on daily life activities, various sensory-based interventions have been developed to restrain the difficulties [28], proving effective in reducing sympathetic responses.

Since this picture, some theoretical accounts suggested that the atypical attentional control classically associated with ASD could be the byproduct of a malfunction in the processing of sensory inputs [29,30]. In this same vein, other authors [15,31,32,33] proposed that early issues in sensory processing, detectable by reports or parents’ interviews, could contribute to refining and possibly anticipating the diagnosis.

A point of interest still under-investigated is the relationship between sensory alterations and cognitive level in developmental age, especially in children with ASD. A viable way to tackle this issue is to match groups according to their mental age, which correlates with the individual cognitive performance. For instance, Baranek and coworkers [34] found that cognitive abilities correlate with the severity of sensory alterations, even if other studies [35] failed to replicate the same results. Overall, the paucity of studies matching participants according to their cognitive levels, the different methods used for this matching, and inconsistent results advocate for more research using such a design.

Starting from these premises, the present study aims to describe the sensory processing patterns in school-aged children with ASD. The Sensory Processing Measure (SPM-2) test was administered to the parents of 105 children with ASD and 70 typically developing peers. The SPM-2 is a norm-referenced measure of function covering multiple sensory modalities, including the visual, auditory, tactile, proprioceptive (body awareness), and vestibular (balance and motion) sensory systems, as well as praxis and social participation. We first compared the scores of the two groups to verify the prevalence of sensory abnormalities in children with ASD. Subsequently, we investigated the role of cognitive level (high- and low-functioning) and explicitly reported sensory symptoms in explaining the scores at the SPM-2. Isolating the specific sensory features correlated with the cognitive level would ultimately bring insights into the causes and consequences of sensory alterations in ASD, potentially enriching the diagnostic and therapeutic procedures with a more comprehensive view of the neurobiology of autism.

## 2. Materials and Methods

### 2.1. Participants

One hundred and seventy-five children were included in the study. The sample was composed of 105 children (17 f, 88 m) that had received a diagnosis of Autism Spectrum Disorders (ASD) (mean age = 9.3 years; SD = 2.0), and 70 (33 f, 37 m) typically developing children (TD) (mean age = 8.8 years; SD = 2.0). Participants with ASD were recruited at the Department of Child Psychiatry and Psychopharmacology of IRCCS Stella Maris and the Department of Clinical and Experimental Medicine of the University of Catania during the period January 2021–July 2021. TD children were recruited in primary schools in Taranto as children matching the ASD group in terms of age. Parents were administered a brief questionnaire relative to their overall health condition and clinical history. Children with a history of neurological or psychiatric disorders were not enrolled, as well as those whose teachers expressed concerns about their development. Clinical and demographic information about the two groups is summarized in Table 1.

For the ASD population, inclusion criteria were: (a) diagnosis of autism spectrum disorder according to DSM-5 criteria [18] and confirmed using algorithm cutoffs on the ADOS-2 [36]; (b) age 6–12 years; (c) lack of comorbidities according to their medical records. Exclusion criteria consisted of (a) presence or history of any other axis I mental disorder; or (b) history of traumatic brain injury or any other neurological disorder in their medical record.

All subjects were native Italian speakers. Informed written consent was obtained from the parents of all participants. The study was approved by the Pediatric Ethics Committee of the Tuscany Region (Approval number: ACCESS 144/21) and was conducted according to both the ethical standards of the Declaration of Helsinki and the Italian Association of Psychology (AIP).

### 2.2. Procedures

**Figure 1:**
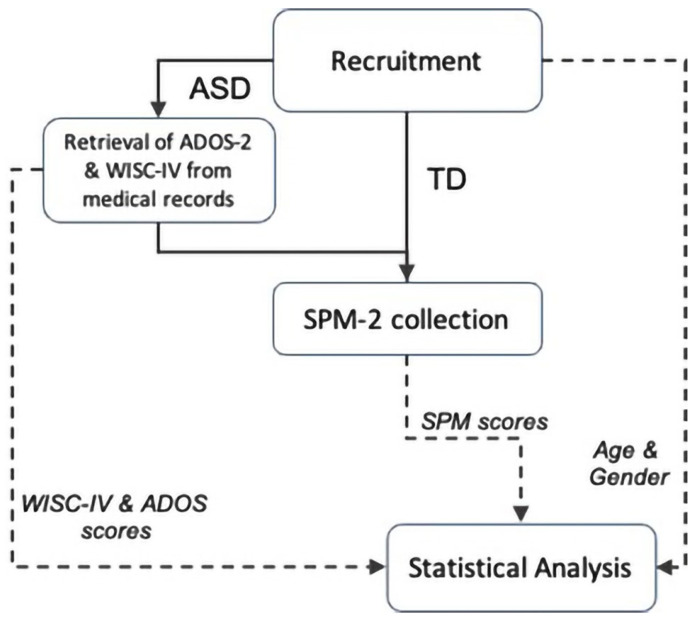
Workflow of the study. Boxes refer to the procedures performed by experimenters. Solid arrows indicate the connection between boxes; the dashed arrows connect the box where an input is generated with the box where that input is used.

The workflow of the study procedures is depicted in Figure 1. Children with ASD were evaluated using clinical tests (ADOS-2 and WISC-IV). The ADOS-2 [36] is a semi-structured, standardized assessment of communication, social interaction, play, and restricted and repetitive behaviors. It provides a highly accurate picture of current symptoms, unaffected by language. The WISC-IV [37] is a scale for assessing cognitive ability, which measures verbal comprehension, perceptual reasoning, working memory, and processing speed. According to their WISC scores, children with ASD were subdivided into two groups, namely high (HF, IQ > 80) and low (LF, IQ < 80) functioning.

In addition, parents of all participants were interviewed using the Sensory Processing Measure (SPM-2) [38]. It requires just 15 to 20 min. The Home Form (adopted for this study) yields eight parallel standard scores: social participation, vision, hearing, touch, body awareness (proprioception), balance and motion (vestibular function), planning and ideas (praxis), and total sensory systems. Raters of the SPM must observe the child in the environment being rated for at least one month, but the child does not need to be present.

### 2.3. Data Analysis

The data analysis comprised two main stages. We applied an ANCOVA to compare the SPM total score and sub-scores between ASD and TD samples in the first one. To this aim, we considered the scores of SPM as the dependent variable and the diagnosis (ASD or not) as the categorical independent variable. The subjects’ gender was also included as a categorical factor, and the age as a covariate. In this way, we tested the main and interacting effects of diagnosis after controlling for the possible effects of age and gender variables.

The second stage aimed at revealing whether the cognitive level of children with ASD affects the SPM scores, after accounting for the fact that both groups include a mixture of children presenting sensory symptoms and children not reporting sensory abnormalities. To account for these factors, we applied an ANCOVA considering the SPM total score and sub-scores as dependent variables, and the functioning (HF and LF) and symptoms (present or not) as categorical independent variables. We also added the ADOS-2 scores as a covariate. This analysis aimed to evaluate the main and interacting effects of functioning and symptoms variables on the scores of SPM after adjusting for the possible effects of ADOS-2 scores. The significance threshold was set at 5%, and in the case of significant effects, post-hoc analysis was Bonferroni-corrected to account for multiple comparisons.

## 3. Results

**Figure 2:**
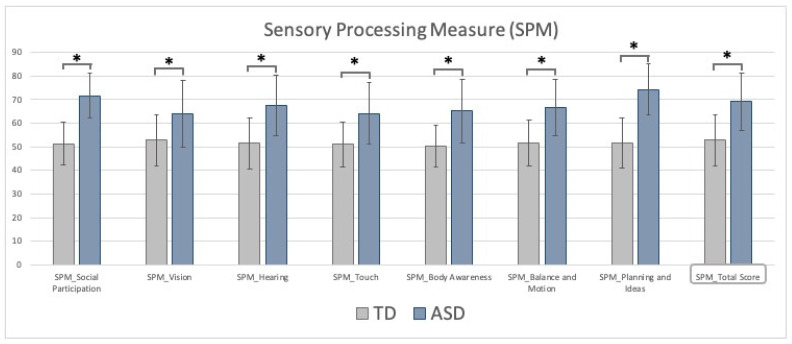
SPM-2 subscale and total score for TD (grey) and ASD (blue) groups. Bars indicate the standard deviations; asterisks indicate the statistical significance.

The contrast between groups indicated that ASD children typically exhibit higher SPM scores, suggesting worse sensory processing, praxis, and social participation in daily activities. After adjusting for the age of the participants, ASD children presented larger and highly significant scores compared to TD for all the SPM subscales (F (1170) > 19, all *p* < 0.001, see Figure 2). No significant effect of gender or age was found for any scores. These results indicate that the sensory processing is altered in ASD children relative to TD peers. Such a difference does not depend on the age of the participants or the gender bias intrinsic to any ASD/TD comparisons.

Examining the SPM total score within the ASD group, the presence of sensory symptoms significantly impacts the SPM (F (1100) = 177.6, *p* < 0.001), with children lacking sensory abnormalities presenting an SPM total score of approximately 22 points lower. On the contrary, no effect emerged for the functioning factor, with HF and LF characterized by very similar scores (F (1100) = 0.32, *p* = 0.57). No functioning x symptoms interaction was found (F (1100) = 0.47, *p* = 0.49). In addition, no significant effect of the ADOS-2 scores was found.

While this picture is true at the global level, one could wonder whether the same pattern applies throughout the multiple subscales of SPM, each reflecting a specific domain. For this reason, the same analysis was applied to the seven SPM subscales (see Table 2).

Not surprisingly, all subscales presented a significant main effect of symptoms (all *p* < 0.002), confirming that children lacking sensory abnormalities have lower scores at all the SPM subscales. No functioning x symptoms interaction was found at any of the subscales (all *p* > 0.2), and the same applies to the systematic lack of significant effects of the ADOS-2. Overall, these results suggest that the ADOS-2 scores and the cognitive level of ASD children, often considered as severity indexes, do not interact with the sensory processing indexed by SPM.

A less stable statistical pattern emerged relative to the main effect of functioning. Social participation (F (1100) = 3.83, *p* = 0.05) and hearing (F (1100) = 4.93, *p* = 0.02) scores presented a significant main effect of functioning, with the scores of LF children exceeding those of HF children by around five points. Although the functioning effect appears milder than the symptoms effect, it was significant for both subscales. No main effect of functioning was found for the vision, touch, body awareness, and planning and ideas subscales. Finally, an unexpected finding emerged for the balance and motion subscale (F (1100) = 6.76, *p* = 0.01). Indeed, a reversed pattern characterized the main effect of functioning, with HF children presenting higher scores.

## 4. Discussion

The present study described the sensory processing abnormalities in school-aged children with ASD compared with typically developing peers. In line with the vast available literature [2,19], children with ASD exhibited a strong prevalence of sensory alterations not only in the SPM-2 total scores but also in the multiple subscales, each addressing specific sensory modalities. While these results were largely expected, at the same time, they indicate that our sample can be considered representative of children with ASD in general.

The major aim of the study was to disentangle within the ASD sample whether the cognitive level and the presence of sensory symptoms explain the scores exhibited at the SPM-2. To this end, we noted whether each child’s medical record included or not the presence of sensory disturbances and labeled each participant as high or low functioning (HF or LF) according to their IQ. Not surprisingly, all the SPM-2 scores presented a significant main effect related to the presence of sensory symptoms. In other words, children suffering from sensory alterations had worse scores at the SPM-2. Even if such a finding may seem trivial, modeling the presence of symptoms within the factorial design is extremely relevant as it allows us to isolate its related variance from those specific to the cognitive factor and the interaction between them.

None of the tested scores showed a significant functioning x symptoms interaction, suggesting a limited interplay between the two variables. More heterogeneous is the pattern of findings that emerges from the main effect of cognitive functioning. Indeed, while HF and LF children did not differ in the SPM-2 total scores, a significant main effect was found for different subscales, namely hearing, social participation, and balance and motion.

Concerning the acoustic domain, children with ASD show delayed evoked neural responses to auditory tones compared with TD [39,40], and this latency predicts their symptom severity [41]. Being observed in response to both pure tones and complex stimuli (such as speech) [40], the hypothesis was advanced that this difference might precipitate higher-order autistic difficulties in communication [42,43]. Comparing HF and LF, previous studies report better auditory capacities in HF [44], who can even surpass non-autistic young adults in terms of unexpected and expected sound detection [45]. On the contrary, the auditory processing in LF could be compromised by a sensory overload, in turn interfering with social communication.

Here, it is important to remark that—along with vision—audition is a cornerstone of social interaction during early life. At the same time, in infants with ASD, atypical sensory development precedes the emergence of social-communicative symptoms [46], leading authors to postulate that sensory impairment might be causal to the social impairment in ASD (the so-called “Sensory-first” accounts [47,48,49]), or at least that the two might interact closely [2].

Considering the above reasoning, it makes sense that a significant effect of functioning was found in the social participation subscale, still presenting better scores for HF. Indeed, this function might be secondarily hindered by primary sensory impairments such as the visual and auditory ones. In addition, the better score for HF might result as a consequence of the camouflage effect described in subjects with a cognitive level near the normal range [49]. The camouflage effect refers to the use of strategies (e.g., reducing repetitive behavior, “stimming”, or responses to sensory over-stimulation) to mask autistic features, mimicking or performing neurotypical peers’ behavior during social interaction. Using these strategies, an individual with ASD forces himself to engage in visual contact or other non-verbal communication skills [50,51].

In summary, the hearing and social participation subscales returned a coherent picture in which autistic children have primary sensory deficits whose severity might, in turn, affect their social skills.

An opposite result concerned the significant main effect of functioning at the balance and motion subscale, in which HF appeared more impaired than LF children. HF children are often clumsy, slower, and less fluent, with impaired gross motor skills [52,53,54]. In the attempt to connect the sensory profile with motor functioning, we must consider that sensory information forms the building blocks for higher-order cognitive functions [55]. Thus, an altered sensory profile could lead to impairments in higher motor functions, such as movement ideation and motor planning [56]. In particular, the balance domain is strictly related to vestibular processing. Vestibular issues are under-reported in children with ASD, less investigated than other sensory abnormalities [57,58], and not considered to be either primary or specific to autism [59]. The paucity of literature on the theme and the marginal statistical effect reported in our study advocate for additional future investigation, and one should be aware that this aspect may be an important ingredient for the clinical picture of children with ASD.

The result that HF are more impaired than LF in the balance and motion subscale must be taken with caution. Its counterintuitive nature requires further confirmations, possibly combining multiple observations and measurements. On the other side, we cannot rule out that some sensory fingerprints related to low-level motor features might be more compromised in the HF phenotype, strengthening the notion of a multifaceted spectrum.

A few limitations must be disclosed examining our study in light of its results. On one side, larger cohorts of ASD would be needed to make reliable but subtle effects emerge and allow researchers to apply stratifications according to the main clinical and demographic variables. On the other side, complementing this questionnaire-based information with biological signals representative of sensory processes could bridge the gap between the notion that a deficit is present and the knowledge of its underlying mechanisms.

## 5. Conclusions

Aside from the classical knowledge that the ASD population suffers from sensory processing disorders, we revealed that different sensory patterns are associated with high or low cognitive functioning. While this aspect does not reflect a different total score at SMP, an HF versus LF difference emerges from the analysis of the individual subscales. This only partial difference might explain the inconsistency of previous findings [34,35]. Beyond its neurobiological interest, such knowledge may be of fundamental importance for the individualization of psychoeducational interventions in both preschool- and school-aged children and later developmental stages. Isolating the specific sensory features correlated with the cognitive level would ultimately bring insights into the causes and consequences of sensory alterations in ASD, potentially enriching the diagnostic and therapeutic procedures with a more comprehensive view of the neurobiology of autism.

## Figures and Tables

**Table 1:** Clinical and demographic features of participants.

		N	Age	Gender	ADOS-2	IQ
TD		70	8.8 ± 2.0	33 f; 37 m		
ASD		105	9.3 ± 2.0	17 f; 88 m	5.8 ± 1.7	88.4 ± 25.5
	HF	66	9.4 ± 2.1	7 f; 59 m	5.4 ± 1.5	105.1 ± 14.7
	LF	39	8.9 ± 1.7	10 f; 29 m	6.6 ± 1.9	60.4 ± 10.4

**Table 2:** Contrast between LF and HF at the SPM-2 subscales and total score. * *p* < 0.05.

	LF	HF	Symptoms	Functioning
Social Participation	74.1 ± 8.5	71.3 ± 9.9	*p* = 0.002 *	*p* = 0.05 *
Vision	63.6 ± 13.3	67 ± 14.7	*p* < 0.001 *	*p* = 0.75
Hearing	68.7 ± 12.1	67.4 ± 13.6	*p* < 0.001 *	*p* = 0.02 *
Touch	60.1 ± 12.2	66.6 ± 13	*p* < 0.001 *	*p* = 0.10
Body Awareness	62 ± 12.9	69.1 ± 13.3	*p* < 0.001 *	*p* = 0.14
Balance and Motion	63.3 ± 12.2	69.6 ± 11.6	*p* < 0.001 *	*p* = 0.01 *
Planning and Ideas	76.2 ± 8.4	73.7 ± 11.8	*p* = 0.002 *	*p* = 0.16
Total	67 ± 11.8	73 ± 12.2	*p* < 0.001 *	*p* = 0.66

## Data Availability

The data presented in this study are available on request from the corresponding author. The data are not publicly available due to ethical restrictions.
